# Rituximab is not a “magic drug” in post-transplant recurrence of nephrotic syndrome

**DOI:** 10.1007/s00431-016-2747-1

**Published:** 2016-06-30

**Authors:** Ryszard Grenda, Wioletta Jarmużek, Jacek Rubik, Barbara Piątosa, Sylwester Prokurat

**Affiliations:** 1 Department of Nephrology, Kidney Transplantation & Hypertension, The Children’s Memorial Health Institute, Warsaw, Poland; 2 Histocompatibility Lab, The Children’s Memorial Health Institute, Warsaw, Poland

**Keywords:** Nephrotic syndrome, Renal transplantation, Rapid recurrence, Rituximab

## Abstract

Pediatric patients with end-stage renal failure due to severe drug-resistant nephrotic syndrome are at risk of rapid recurrence after renal transplantation. Treatment options include plasmapheresis, high-dose of cyclosporine A/methylprednisolone and more recently—rituximab (anti-B CD_20_ monoclonal depleting antibody). We report five patients with immediate (1–2 days) post-transplant recurrence of nephrotic syndrome, treated with this kind of combined therapy including 2–4 weekly doses of 375 mg/m^2^ of rituximab. Only two (of five) patients have showed full long-term remission, while the partial remission was seen in two cases, and no clinical effect at all was achieved in one patient. The correlation between B CD_19_ cells depletion and clinical effect was present in two cases only. Severe adverse events were present in two patients, including one fatal rituximab-related acute lung injury.

*Conclusion*: The anti-CD_20_ monoclonal antibody may be not effective in all pediatric cases of rapid post-transplant recurrence of nephrotic syndrome, and benefit/risk ratio must be carefully balanced on individual basis before taking the decision to use this protocol.

**Table:** 

**What is Known:** • *nephrotic syndrome may recur immediately after renal transplantation* • *plasmapheresis combined with pharmacotherapy is used as rescue management* • *rituximab was reported as effective drug both in primary and post-transplant nephrotic syndrome*
**What is New:** • *rituximab may not be effective is several cases of post-transplant nephrotic syndrome due to variety of underlying mechanisms of the disease, which may be or not be responsive to this drug* • *there may be no correlation between drug-induced depletion of specific B cells and clinical effect; this might suggest B-cell independent manner of rituximab action*

## Introduction

The recurrence of primary renal disease after renal transplantation is a risk factor of inferior outcome [23]. Drug-resistant nephrotic syndrome leading to end-stage renal failure is one the most aggresive glomerular diseases recurring after renal transplanation [1]. Immediate recurrence suggests the role of preformed and circulating protein permeability factor(s), which bind to specific targets in transplanted kidney. Identification of responsible factor(s) is an ongoing subject of clinical investigations for years, and several hypotheses have been raised, including protein permeability factor suggested by Savin’s group [21], soluble urokinase type plasminogen activator receptor (suPAR) [24], cardiothropin-like cytokine 1(CLC-1) [13], and anti-CD_40_ antibody [4]. The hypothesis of circulating factor(s)-related mechanism of post-transplant recurrence of nephrotic syndrome is translated into therapeutic protocols, including plasmapheresis (to remove the factor(s)) [17, 18], corticosteroids (to stabilize the cytoskeleton of podocytes and reduce podocytes apoptosis) [19], cyclosporine A (to stabilize the cytoskeleton of podocytes) [5], and rituximab (monoclonal antibody depleting B CD_20_ cells) to deplete the cytokines-producers [7, 15]. There are conflicting data on efficacy of rituximab used for treatment of post-transplant recurrence of nephrotic syndrome in adult and pediatric patients. The purpose of our study was to present the summary of our experience with the efficacy and safety of rituximab in five children after first renal transplantation, who presented rapid recurrence of nephrotic syndrome.

## Patients and methods

A retrospective chart review was performed and included five children at the age from 5 to 12 years (at the day of renal transplantation), who presented immediate (day 1 or 2) recurrence of nephrotic syndrome (NS) post-transplant. The criterion of chart selection was the use of rituximab for the treatment of NS recurrence after transplantation. The drug-resistant nephrotic syndrome was the underlying cause of end-stage renal disease. Focal segmental glomerulosclerosis (FSGS) was present in native renal biopsy in two patients, mesangial-proliferative glomerulonephritis (MesPGN) in two, and minimal change nephrotic syndrome (MCNS) in one case. One patient had confirmed heterozygous podocine (NPHS2) genetic mutation (p.R229Q). There was 3 to 8 years interval between introducing dialysis and primary renal transplantation. There were four deceased donor-related and one living-related transplantation performed, the last one in a patient with genetic podocine mutation (patient’s father was a donor). All patients received triple immunosuppression (CsA + MMF + Pred). All five demonstrated immediate recurrence of nephrotic syndrome (four on the day 1 and one on the second day post-transplant), with gross proteinuria and oliguria. Plasmapheresis was introduced in all five patients, and in four cases, rituximab was directly added to the protocol at the dose of 375 mg/m^2^ on weekly basis. In one case, the introduction of rituximab was delayed due to primary promising effect of plasmapheresis; however, once the patient became plasmapheresis-dependent, the drug was added during fourth month post-transplant [8]. Four patients received four weekly doses, and in one case the drug administration was limited to two doses due to severe relapsing infections The count of B CD_19_ cells was monitored on weekly and then monthly basis by flow cytometry. The clinical characteristics and treatment course in summarized in Table 1. The complete remission was achieved in two patients (no. 1 and no. 3), with complete depletion of B CD_19_ for up to 4 and 2 months (after last dose of rituximab), respectively, and with no adverse drug-related adverse events. The remission was sustained within 7 and 5 years of follow-up, respectively, with no specific treatment beyond standard triple immunosuppression (CsA + MMF + Pred). Only partial remission (significant, however floating proteinuria; from 0.4 to 3.5 g and from 0.5 to 2.0 g/24 hours; respectively) was seen in two cases (no. 4 and no. 5), presenting complete B CD_19_ depletion up to 7 and 4 months after treatment, respectively. Serious adverse events were present in these two cases, including relapsing severe infections (blood culture was positive for Klebsiella pneumoniae) (case no. 4), which limited the number of scheduled rituximab doses to 2 and severe acute lung injury (RALI) (case no. 5). Patient no. 4 lost the graft 2.5 years after transplantation due to chronic nephropathy. Patient no. 5 died due to non-infectious rapid lung fibrosis. There was no therapeutic effect at all in the case no. 2, who was also finally given oral galactose; however, the graft was never functioning and was removed 7 months post-transplant.

**Table 1:** Clinical chacteristics and treatment course of five children with post-transplant recurrence of nephrotic syndrome

Patient no.	Age at diagnosis of NS (years), gender renal biopsy	Age at trans-plantation (years) origin of the graft	Timing of NS recurrence post-transplant (days)	Treatment day of rituximab introduction after transplantation	Duration of B CD19 depletion (months)/correlation of number of B CD_19_ cells to clinical effect	Clinical effects renal graft biopsy	SAE
1	2, male FSGS	5.5 deceased donor	1	PF + CsA + MMF + MP + rituximab 4 × 375 mg/m^2^ day 133	4 Yes	Complete remission (7 years of follow-up) MCNS	No
2	2, female MCNS	5 deceased donor	1	PF + CsA + MMF + MP + rituximab 4 × 375 mg/m^2^ + galactose day 25	3 No	Failure; graft removed 7 months later FSGS	No
3	2, female MesPGN (NPHS2 gene mutation-single heterozygous mutation; p.R229Q)	10 living-related donor	1	PF + CsA + MMF + MP + rituximab 4 × 375 mg/m^2^ day 15	2 Yes	Complete remission (5 years of follow-up) FSGS	No
4	6, female FSGS	12 deceased donor	1	PF + CsA + MMF + MP + rituximab 2 × 375 mg/m^2^ day 21	7 no	Partial remission; graft removed 2.5 years later FSGS	Relapsing severe infections
5	6, male MesPGN	10 deceased donor	2	PF + CsA + MMF + MP + rituximab 4 × 375 mg/m^2^ day 15	4 no	Partial remission MPGN	RALI (fatal)

*MesPGN* mesangial-proliferative glomerulonephritis, *FSGS* focal segmental glomerulosclerosis, *MCNS* minimal change nephrotic syndrome, *NPHS2* podocine, *CsA* cyclosporine A, *MMF* mycophenolate mofetil, *MP* methylprednisolone, *PF* plasmapheresis, *SAE* serious adverse events, *RALI* rituximab-related acute lung injury

## Discussion

Rituximab has been used for treatment of post-transplant nephrotic syndrome for a decade, and initial reports were in favor to the drug efficacy [7, 12, 16]. Our preliminary experience (case no. 1) was also very positive [8] and encouraged us to use this protocol. The reports published later on have revealed the conflicting data on clinical efficacy. Published 13 studies (mainly case reports or case series reports) included overall 44 pediatric patients treated with rituximab for post-transplant recurrence of nephrotic and among those 27 patients (55 %) presented complete remission, 14 (28.5 %) partial remission of nephrotic syndrome, and no effect was present in the remaining 8 (16.5 %) cases [14].

### Rituximab was always used in combination with plasmapheresis and other drugs

It must be noted that most of the reported patients, treated for recurrence of nephrotic syndrome after renal transplantation with rituximab, underwent the combined therapy with plasma exchange and post-transplant triple immunosuppression including calcineurine inhibitors, steroids, and antiproliferative drugs, so the final clinical effect (if positive) might be the summary result of several therapeutic mechanisms. Moreover, the timing of introduction and the number of rituximab doses used in this setting was variable, as the drug was given also preemptively (as prophylaxis) at the day of transplantation [6] or as rescue therapy of overt recurrence of nephrotic syndrome [7, 12, 16, 22]. The number of doses ranged from single to six weekly doses of 375 mg/m^2^. The correlation between B CD_20_/CD_19_ cells depletion and clinical effect was also variable [14]. Among patients described in our report, four (out of five) have received rituximab as first-line therapy, and in remaining one, the drug was prescribed as second-line treatment due to persistent dependency from plasmapheresis. In four of our cases, four fixed weekly doses of 375 mg/m^2^ were given, and in remaining one, the number of scheduled doses was limited to two, due to relapsing infections. It is not clear whether the time interval (shorter versus longer) between post-transplant NS recurrence and introduction of rituximab is important for the overall efficacy of this intervention. Some authors expressed such suggestion in the discussion of their reports; however, there is no evidence so far. Almost universally, rituximab therapy was combined with plasmapheresis, and other suggestions stated that rituximab should be introduced early after the evidence of plasmapheresis failure [10, 12, 22].

### Why the efficacy of rituximab is variable

It is not clear, whether the lack of significant clinical effect in three (of five) of our cases is related to specificity of this particular setting of the patients, in whom the recurrence was immediate (day 1 or 2), which may suggest the high concentration/activity of hypothetic circulating factors. Unfortunately, at the time of this treatment the evaluation of suPAR concentration in serum or urine was not available. The clinical course of these cases also show that long waiting time on dialysis (from 3 to 8 years) has no protective effect in terms of risk of rapid recurrence of nephrotic syndrome, as was carefully suggested based on some clinical observations [2]. Using similar approach, we faced two successful and three disappointing cases. B cells depletion was achieved in all cases, irrespective from the clinical outcome, so apparently rituximab was effective in terms of pharmacodynamics activity, independent from the absence of clinical effect in those patients, who did not respond. This might correspond with the data on B cell-independent mechanism of rituximab, which may act via regulation (preservation) of sphingomyelin phosphodiesterase acid-like 3b (SMLPD-3b), a protein which is involved in the podocyte cytoskeleton activity [6]. The anecdotal use of galactose in case no. 2, based on hypothesis of local inhibition of protein permeability factor in podocytes by galactose, was not effective either [20, 21]. Probably achieving (or not) the complete remission in particular cases is the effect of random targeting (or not) the patient, in whom the mechanism of nephrotic syndrome is directly related to abnormal activity of B cells. It is also possible that partial efficacy is related to B-independent manner of rituximab action [6]. Interestingly, the complete depletion of B cells was also present in our case no. 2, who showed complete resistance to rituximab, suggesting that there are specific cases, in whom neither B cell-dependent nor B cell-independent (local) mechanisms of the drug are relevant to reduce heavy post-transplant proteinuria. This is possible that this patient had T cell-dependent mechanism of the disease; however, plasmapheresis, which theoretically should be helpful in removing T cell driven cytokines (such as IL-13 stimulating local expression of CD80 molecule in podocytes) [3], was not effective either. Current experience on potential of use of abatacept (specific inhibitor of CD80) in post-transplant recurrence of NS, resistant to plasmapheresis, and rituximab [25] was not known at the time when our case was treated.

Unfortunately, we are still at an early stage of distinguishing the different patterns of the mechanisms of post-transplant nephrotic syndrome; however, recent data on the use of diagnostic tool to identify patients at high risk of FSGS recurrence are encouraging [4].

One of our patients had heterozygous mutation of podocine and received living-related transplant. There were some reports on recurrence of nephrotic syndrome after renal transplantation in children with podocine genetic mutations, with high variety in terms of post-transplant time to proteinuria (range 4 to 10 years). The final outcome of reported six cases was good [11]. As the recurrence was immediate (first day) in our case, we presume that she presented combined, genetic defect and immunological injury, and the latter one was the major cause of rapid recurrence.

### Safety concern

Another issue is safety of this protocol. We have faced two cases of severe adverse events, including severe recurrent bacterial infections in case no. 4 suggesting overimmunosuppression. The most significant adverse event was RALI (rituximab-associated acute lung injury) in case no. 5, which was reported in details as case report [9]. The risk/benefit ratio is then individual and must be taken into consideration.

In summary, we present disappointing results of rituximab plasmapheresis-based rescue protocol of treating immediate recurrence of post-transplant nephrotic syndrome. It is unclear, whether aggressive pattern of immediate recurrence was the reason of the treatment failure in three cases. Center experience was the reason of the evolution of our attitude in terms of using this protocol, from enthusiastic to reluctant.

## Abbreviations

B CD_19_, B CD_20_: Lymphocytes B expressing specific receptors bound by monoclonal antibody (rituximab)
CsA: Cyclosporine A
MMF: Mycophenolate mofetil
MP: Methylprednisolone
PF: Plasmapheresis
NS: Nephrotic syndrome
FSGS: Focal segmental glomerulosclerosis
MCNS: Minimal change nephrotic syndrome
MesPGN: Mesangial-proliferative glomerulonephritis
IL-13: Interleukin 13
CD80: T-cell co-stimulatory molecule
